# Supplementary material for: Expression of SDF-1/CXCR4 and related inflammatory factors in sodium fluoride-treated hepatocytes
Source: PLoS One. 2024 Jun 21;19(6):e0302530. doi: 10.1371/journal.pone.0302530 (PMC11192373; doi:10.1371/journal.pone.0302530)
Supplement: Attachment — Submitted filename: PONE-D-23-32257_reviewer Khalil.pdf [file pone.0302530.s002.pdf]

PLOS ONE

Expression of SDF-1/CXCR4 and Related Inflammatory Factors in Sodium Fluoride-Treated Hepatocytes

--Manuscript Draft--

**Manuscript Number:**	PONE-D-23-32257
**Article Type:**	Research Article
**Full Title:**	Expression of SDF-1/CXCR4 and Related Inflammatory Factors in Sodium Fluoride-Treated Hepatocytes
**Short Title:**	Expression of SDF-1/CXCR4 and Related Inflammatory Factors in Sodium Fluoride-Treated Hepatocytes
**Corresponding Author:**	Qiang Zhang<br>Qinghai University<br>西宁市, ---选择一个--- CHINA
**Keywords:**	LX-2; fluoride; SDF-1/CXCR4; Inflammatory Factors; liver
**Abstract:**	Abstract: At present, the mechanism of fluorosis-induced damage to the hepatic system is unclear. Studies have shown that excess fluoride causes some degree of damage to the liver, including inflammation. The SDF-1/CXCR4 signaling axis has been reported to have an impact on the regulation of inflammation in human cells. In this study, we investigated the role of the SDF-1/CXCR4 signaling axis and related inflammatory factors in fluorosis through in vitro experiments on human hepatic astrocytes (LX-2) cultured with sodium fluoride. CCK-8 assays showed that the median lethal dose at 24 h was 2 mmol/l NaF, and these conditions were used for subsequent enzyme-linked immunosorbent assays (ELISAs) and quantitative real-time polymerase chain reaction (qPCR) analysis. The protein expression levels of SDF-1/CXCR4 and the related inflammatory factors nuclear factor-κB (NF-κB), interleukin-6 (IL-6), tumor necrosis factor-α (TNF-α) and interleukin 1β (IL-1β) were detected by ELISAs from the experimental and control groups. The mRNA expression levels of these inflammatory indicators were also determined by qPCR in both groups. Moreover, the expression levels of these factors were significantly higher in the experimental group than in the control group at both the protein and mRNA levels ($P < 0.05$). Excess fluorine may stimulate the SDF-1/CXCR4 signaling axis, activating the inflammatory NF-κB signaling pathway and increasing the expression levels of the related inflammatory factors IL-6, TNF-α and IL-1β. The identification of this mechanism is important for elucidating the pathogenesis of fluorosis-induced liver injury.
**Order of Authors:**	瑞 杨
	Hongting Shen
	MingJun Wang
	YaQian Zhao
	Shiling Zhu
	Hong Jiang
	Yanan Li
	Guanglan Pu
	Xun Chen
	Ping Chen
	Qing Lu
	Jing Ma
	Qiang Zhang
**Additional Information:**	

Powered by Editorial Manager® and ProduXion Manager® from Aries Systems Corporation

Question	Response
**Financial Disclosure**<br><br>Enter a financial disclosure statement that describes the sources of funding for the work included in this submission. Review the submission guidelines for detailed requirements. View published research articles from *PLOS ONE* for specific examples.<br><br>This statement is required for submission and **will appear in the published article** if the submission is accepted. Please make sure it is accurate.<br><br>**Unfunded studies**<br>Enter: *The author(s) received no specific funding for this work.*<br><br>**Funded studies**<br>Enter a statement with the following details:<br>• Initials of the authors who received each award<br>• Grant numbers awarded to each author<br>• The full name of each funder<br>• URL of each funder website<br>• Did the sponsors or funders play any role in the study design, data collection and analysis, decision to publish, or preparation of the manuscript?<br>• **NO** - Include this sentence at the end of your statement: *The funders had no role in study design, data collection and analysis, decision to publish, or preparation of the manuscript.*<br>• **YES** - Specify the role(s) played.<br><br>* typeset	Conceptualization, R.Y.; methodology, R.Y.; software, R.Y.; validation, R.Y. and H.S.; formal analysis,R.Y. and H.S.; investigation, R.Y., G.P. and R.Y.; data curation, Y.L., J.H. and M.W.; writing—original draft preparation, R.Y; writing—review and editing, R.Y.; visualization, R.Y., X.C. and P.C. and J.M. and L.Q.; supervision,R.Y. and Y.Z. and S.Z.; project administration, Q.Z.; funding acquisition, Q.Z. All authors have read and agreed to the published version of the manuscript.
**Competing Interests**<br><br>Use the instructions below to enter a competing interest statement for this submission. On behalf of all authors, disclose any competing interests that could be perceived to bias this work—acknowledging all financial support and any other relevant financial or non-financial competing interests.	The authors have declared that no competing interests exist.

This statement is required for submission and **will appear in the published article** if the submission is accepted. Please make sure it is accurate and that any funding sources listed in your Funding Information later in the submission form are also declared in your Financial Disclosure statement.<br><br>View published research articles from *PLOS ONE* for specific examples.<br><br>**NO authors have competing interests**<br><br>Enter: *The authors have declared that no competing interests exist.*<br><br>**Authors with competing interests**<br><br>Enter competing interest details beginning with this statement:<br><br>*I have read the journal's policy and the authors of this manuscript have the following competing interests: [insert competing interests here]*<br><br>* typeset	
**Ethics Statement**<br><br>Enter an ethics statement for this submission. This statement is required if the study involved:<br><br>• Human participants<br>• Human specimens or tissue<br>• Vertebrate animals or cephalopods<br>• Vertebrate embryos or tissues<br>• Field research<br><br>Write "N/A" if the submission does not require an ethics statement.<br><br>General guidance is provided below. Consult the submission guidelines for detailed instructions. **Make sure that all information entered here is included in the Methods section of the manuscript.**	NO

**Format for specific study types**<br><br>**Human Subject Research (involving human participants and/or tissue)**<br>• Give the name of the institutional review board or ethics committee that approved the study<br>• Include the approval number and/or a statement indicating approval of this research<br>• Indicate the form of consent obtained (written/oral) or the reason that consent was not obtained (e.g. the data were analyzed anonymously)<br><br>**Animal Research (involving vertebrate animals, embryos or tissues)**<br>• Provide the name of the Institutional Animal Care and Use Committee (IACUC) or other relevant ethics board that reviewed the study protocol, and indicate whether they approved this research or granted a formal waiver of ethical approval<br>• Include an approval number if one was obtained<br>• If the study involved *non-human primates*, add *additional details* about animal welfare and steps taken to ameliorate suffering<br>• If anesthesia, euthanasia, or any kind of animal sacrifice is part of the study, include briefly which substances and/or methods were applied<br><br>**Field Research**<br><br>Include the following details if this study involves the collection of plant, animal, or other materials from a natural setting:<br>• Field permit number<br>• Name of the institution or relevant body that granted permission	
**Data Availability**<br><br>Authors are required to make all data underlying the findings described fully available, without restriction, and from the time of publication. PLOS allows rare exceptions to address legal and ethical concerns. See the PLOS Data Policy and FAQ for detailed information.	Yes - all data are fully available without restriction

Powered by Editorial Manager® and ProduXion Manager® from Aries Systems Corporation

A Data Availability Statement describing where the data can be found is required at submission. Your answers to this question constitute the Data Availability Statement and **will be published in the article**, if accepted.<br><br>**Important:** Stating 'data available on request from the author' is not sufficient. If your data are only available upon request, select 'No' for the first question and explain your exceptional situation in the text box.<br><br>Do the authors confirm that all data underlying the findings described in their manuscript are fully available without restriction?	
**Describe where the data may be found in full sentences. If you are copying our sample text, replace any instances of XXX with the appropriate details.**<br><br>• If the data are **held or will be held in a public repository**, include URLs, accession numbers or DOIs. If this information will only be available after acceptance, indicate this by ticking the box below. For example: *All XXX files are available from the XXX database (accession number(s) XXX, XXX.).*<br>• If the data are all contained **within the manuscript and/or Supporting Information files**, enter the following: *All relevant data are within the manuscript and its Supporting Information files.*<br>• If neither of these applies but you are able to provide **details of access elsewhere**, with or without limitations, please do so. For example:<br><br>*Data cannot be shared publicly because of [XXX]. Data are available from the XXX Institutional Data Access / Ethics Committee (contact via XXX) for researchers who meet the criteria for access to confidential data.*<br><br>*The data underlying the results presented in the study are available*	All relevant data are within the manuscript and its Supporting Information files

from (include the name of the third party and contact information or URL).<br>• This text is appropriate if the data are owned by a third party and authors do not have permission to share the data.<br><br>* typeset	
Additional data availability information:	Tick here if the URLs/accession numbers/DOIs will be available only after acceptance of the manuscript for publication so that we can ensure their inclusion before publication.

Powered by Editorial Manager® and ProduXion Manager® from Aries Systems Corporation

Manuscript

Click here to access/download;Manuscript;Expression of SDF1CXCR4 and Related I(7).docx

Expression of SDF-1/CXCR4 and Related Inflammatory Factors in Sodium Fluoride-Treated Hepatocytes

Rui Yang[1,2], Hongting Shen[2], Mingjun Wang[2], Yaqian Zhao[1,2], Shiling Zhu[1,2], Hong Jiang[2], Yanan Li[2], Guanglan Pu[2], Xun Chen[2], Ping Chen[2], Qing Lu[2], Jing Ma[2], Qiang Zhang [2,]*

[1] Department of Public Health, Medical College, Qinghai University, Xi'ning 810016, China;

[2] Department of Endemic Disease Prevention and Control, Qinghai Institute for Endemic Disease Prevention a nd Control, Xi'ning 811602, China

* Correspondence: wdrr@163.com; Tel.: 0971-6510183

Abstract: At present, the mechanism of fluorosis-induced damage to the hepatic system is unclear. Studies have shown that excess fluoride causes some degree of damage to the liver, including inflammation. The SDF-1/CXCR4 signaling axis has been reported to have an impact on the regulation of inflammation in human cells. In this study, we investigated the role of the SDF-1/CXCR4 signaling axis and related inflammatory factors in fluorosis through in vitro experiments on human hepatic astrocytes (LX-2) cultured with sodium fluoride. CCK-8 assays showed that the median lethal dose at 24 h was 2 mmol/l NaF, and these conditions were used for subsequent enzyme-linked immunosorbent assays (ELISAs) and quantitative real-time polymerase chain reaction (qPCR) analysis. The protein expression levels of SDF-1/CXCR4 and the related inflammatory factors nuclear factor-κB (NF-κB), interleukin-6 (IL-6), tumor necrosis factor-α (TNF-α) and interleukin 1β (IL-1β) were detected by ELISAs from the experimental and control groups. The mRNA expression levels of these inflammatory indicators were also determined by qPCR in both groups. Moreover, the expression levels of these factors were significantly higher in the experimental group than in the control group at both the protein and mRNA levels ($P < 0.05$). Excess fluorine may stimulate the SDF-1/CXCR4 signaling axis, activating the inflammatory NF-κB signaling pathway and increasing the expression levels of the related inflammatory factors IL-6, TNF-α and IL-1β. The identification of this mechanism is important for elucidating the pathogenesis of fluorosis-induced liver injury.

Keywords: LX-2; fluoride; SDF-1/CXCR4; inflammatory factors; liver

1. Introduction

Fluorosis affects not only the liver system but also other systems of the body. Different types of fluorosis cause different types of damage to the human body, which has been reported in domestic and international studies. Dental fluorosis was observed in some areas of Mexico as early as 1888, but it was not until 1931 that the link between fluoride in drinking water and dental fluorosis was uncovered [1]. Tooth development is closely related to the functions of enamel-forming cells, and Bronckers [2] concluded that high levels of fluoride can directly affect osteoblasts. In China, endemic fluorosis mainly includes three types of fluorosis: drinking-water fluorosis, coal-burning fluorosis and tea-drinking fluorosis [3]. There are approximately 60 tea-producing countries in the world, and the number of tea drinkers exceeds 2 billion [4]. In contrast, drinking water fluorosis is still one of the most severely damaging types of fluorosis in China [5]. In highly fluoridated areas, local populations have experienced some degree of bone damage due to drinking water with a high fluoride content [6]. Coal-burning fluorosis is also common in China, mainly in the southwest [7,8]. Approximately 43% of the districts and counties in Guizhou Province of China were reported to be impacted by fluorosis, and more than half of the individuals in these areas suffer from fluorosis [9]. With the global economic development and changes in industry and lifestyle, the prevalence of endemic fluorosis has been significantly reduced, but groundwater fluoride contamination and tea-drinking fluorosis are still prevalent in some areas [10-13]. Thus, fluorosis remains a global public health problem that cannot be ignored, and effective control of fluorosis is important for improving quality of life.

Fluorine is an essential trace element that often exists in natural compounds, and it can be bioactive in humans. The intake of an appropriate amount of fluorine is beneficial, for example, for caries prevention and treatment [14]. Excessive intake of elemental fluoride, however, can cause damage to bones and other important organs in the body [15], such as the cardiovascular and hepatic systems and teeth [16-19]. According to epidemiological investigations and in vitro and in vivo experimental studies, fluorosis may cause a certain degree of damage to the liver, inducing hepatic glucose and lipid metabolic dysfunction, and at the cellular level, fluorosis can cause swelling of the endoplasmic reticulum and mitochondria, reduced nuclear volume, nuclear membrane wrinkling and other defects in hepatocytes [20,21]. Fluorosis was shown to increase the levels of inflammatory factors [22]. However, the pathogenesis of the resulting liver inflammation is unclear.

Currently, signaling pathways are a hot topic in the study of the pathogenesis of fluorosis. The stromal cell-derived factor 1 (SDF-1) and chemokine receptor 4 (CXCR4) signaling axis is important for regulating inflammation in the human body [23-25]. CXCR4 is a member of the G protein-coupled receptor superfamily and, along with its specific ligand SDF-1, also known as C-X-C motif chemokine ligand 12, forms a chemokine network that is involved in physiological processes such as cellular immunity, inflammatory cell metastasis, and cell proliferation in humans. Chemokines are a large group of small molecule inflammatory cytokines with molecular weights in the range of 8–12 kDa that act by binding to the corresponding G-protein-coupled seven-transmembrane receptors present on the surface of the target cells to perform various physiological functions [26-28]. The expression of SDF-1/CXCR4 signaling axis members has been reported to be significantly higher in the serum of

patients with osteofluorosis than in that of controls, and the expression level of the inflammatory factor NF-κB was positively correlated with chemokine levels [29]. Activation of the NF-κB pathway is closely linked to inflammatory mechanisms in disease [30-32]. The typical NF-κB pathway induces the production of proinflammatory cytokines such as TNF-α and IL-1β in the innate immune system to mediate inflammatory responses. In addition, the SDF-1/CXCR4 signaling axis was shown to activate the NF-κB signaling pathway to participate in the inflammatory response [33-35], suggesting that the SDF-1/CXCR4–NF-κB signaling pathway may play a role in the pathogenesis of fluorosis. Both the SDF-1/CXCR4 signaling axis and the NF-κB signaling pathway can recruit and mobilize inflammatory factors, which may act as signature downstream signaling molecules [36-38]. The mechanism of the SDF-1/CXCR4 signaling axis and its related inflammatory factors NF-κB, IL-6, IL-1β and TNF-α in the context of the pathogenesis of fluorosis has been poorly studied, and differences in the expression of the above proteins have been reported only in serum samples from individuals in areas with fluorosis [29]. Therefore, the present study aimed to further investigate the role of the CXCL12/CXCR4 signaling axis and the related inflammatory factors IL-1β, TNF-α, IL-6 and NF-κB in the mechanism of fluorosis-induced liver injury at the cellular level.

Although inflammatory damage has not been conclusively established in the pathogenesis of fluorosis, several studies have shown a correlation between these factors. Wheth[illegible] inflammatory factors such as SDF-1/CXCR4, IL-6, TNF-α, NF-κB, and IL-1β play a role in the hepatic system due to fluorosis deserves further exploration.

In this s[illegible]y, LX-2 cells were cultured with sodium fluoride for in vitro experiments, and cell survival was evaluated by the CCK-8 method. HE staining was used to observe the growth and morphology of the cells in each group, and the expression levels of SDF-1 and CXCR4, as well as the inflammatory factors related to the SDF-1/CXCR4 signaling pathway, such as IL-6, TNF-α, NF-κB, and IL-1β, were analyzed to provide evidence for the mechanism of fluoride intoxication-induced liver inflammation. The results provide a basis for the mechanism of hepatic inflammation caused by fluorosis.

2. Materials and Methods

2.1. Cell Culture

Human liver astrocytes (LX-2, 1 vial, model T25) were purchased from Wuhan Procell Life Sciences Co. The cells were cultured in a humidified incubator at 37°C with 5% CO_2 for 48 h. When the cell confluence was greater than 80%, the cells were cultured in a humidified incubator at 37°C with 5% CO_2 and rinsed twice with 2 ml of 1x PBS (Thermo Fisher Scientific, China). Then, 1 ml of trypsin-EDTA (Thermo Fisher Scientific, China) was added to digest the adherent cells, and the cells were placed in a 37°C and 5% CO_2 incubator for 3 min. Then, 4 ml of LX-2 medium was added to the T25 cell culture flasks (Wuhan Procell Life Sciences Co., Ltd., China) to terminate tryptic digestion and mixed with a sterile pasteurized pipette to resuspend the cells. Then, the cells were transferred to 15 ml sterile and enzyme-free centrifuge tubes (Thermo Fisher Scientific, Ltd., China), centrifuged at 1200 × g for 3 min, and resuspended in an appropriate volume of medium for transfer to new culture flasks incubated at 37°C and 5%

CO_2 to continue the cultivation of the cell lines. The present study used cells from the 3rd–7th passages.

2.2. Sodium Fluoride for Cell Treatments

Sodium fluoride (10.5 g, Tianjin Yong da Chemical Reagent Co., Ltd., China) was dissolved in 500 ml of 1x PBS solution and filtered through a membrane to remove any bacteria, and the concentration was adjusted to 500 mmol/l. Complete media with different NaF concentrations were prepared according to each group, as shown in Table 1. After LX-2 cells were cultured at 37°C in a 5% CO_2 incubator for 24 h, the cells were treated with fluorine (n=4).

Table 1. Configuration Table for Different NaF Concentrations

NaF concentration (mmol/l)	LX-2 special medium content (ml)	Amount of 500 mmol/l NaF added (μl)	Total volume (ml)
0.5	9.99	10	10
1	9.98	20	10
2	9.96	40	10
4	9.92	80	10
8	9.84	160	10

2.3. CCK-8 Assay for Cell Viability

Cells (Nanjing Novozymes Bioscience and Technology Co., Ltd., China) were aliquoted into 96-well plates containing 2,000 cells/ml and incubated for 24 h, and the culture medium was poured off when the cell wall density was greater than 80%. The cells were rinsed twice with PBS, and a blank group, control group (0 mmol/l NaF), and experimental groups with different concentrations of sodium fluoride (0.5, 1, 2, 4, 8 mmol/l NaF) were prepared. For each well, except those for the blank group, 200 μl of cell culture medium was added, and the cells were incubated for 12 h, 24 h or 48 h. Then, 10 μl of CCK-8 reagent was added, and the cells were incubated for 1 h with protection from light. A fully automated enzyme labeling instrument (Thermo Fisher Scientific, China) was used to detect the OD value of each group at 450 nm, and cell viability (%) was calculated as (OD of the experimental wells-OD of the blank wells/OD of the control wells) × 100% (n=4).

2.4. HE Staining

LX-2 cell cultures were fixed with 4% paraformaldehyde for 5–10 min, washed with water for 1 min, treated with HE Constant Staining Pretreatment Solution for 1 min, and sequentially stained with appropriate amounts of hematoxylin and eosin staining solution. Finally, the cells were fixed with neutral resin, and the morphology of LX-2 hepatocytes was observed under a light microscope.

2.5. Enzyme-Linked Immunosorbent Assays (ELISAs) of the Expression of Each Target Protein in the Cell Supernatant and Cell Extract of Each Group of Cells

CXCR4, SDF-1, IL-1β, TNF-α, IL-6 and NF-κB ELISA kits were obtained from Nanjing Boyan Biotechnology Co., Ltd. (China). Cell supernatant collection: Cell supernatants were collected by centrifugation of each test group at 20 min (3000 r/min) and stored at -20°C to avoid repeated freezing and thawing. Cell extract collection: adherent cells were washed with PBS at 4°C and digested with trypsin, and then, the cells were collected after 5 min (1000 r/min) and washed with PBS 3 times. Then, 200 μl of 1x PBS was added to resuspend the cells, the cells were ruptured by repeated freezing and thawing, and the supernatant was collected for evaluation. The expression level of each target protein in the cell supernatant was detected by an enzyme labeling instrument (450 nm). The kits were left at room temperature for 30 min before use. For the wash buffer, the concentrate in the kit was diluted with distilled water at 1:20.

All reagents and samples were used at room temperature. Blank wells (no sample or standard added), standard wells (50 μl of standards at different concentrations) and sample wells (50 μl of samples to be tested) were prepared, and 2 replicate wells were prepared for each group. Horseradish peroxidase (HRP)-labeled antibody (100 μl) was added to each of the wells. The plate was sealed using plate sealing film and incubated for 60 min at 37°C in a water bath or thermostat. The liquid was discarded, 300 μl of wash solution was added to each well, and the plate was washed for 20 s and patted dry on absorbent paper; the washes were repeated 5 times. Then, 50 μl each of chromatography substrates A and B was added to each well and incubated at 37°C for 15 min. Finally, 50 μl of termination solution was added to each well, the color changed from blue to yellow, and readings were taken within 15 min.

2.6. RNA Extraction

Total RNA was extracted with a Total RNA Extraction Kit (All Specialty Gold Biotechnology Co., Ltd., Beijing, China). Then, cDNA was synthesized with a Reverse Transcription Kit (All Specialty Gold Biotechnology Co., Ltd., Beijing, China) for gene fragment detection; gene fragments were amplified, and the optimal primers as determined by the HIFI RCR enzyme (All Specialty Gold Biotechnology Co., Ltd., Beijing, China) and HIFI RCR enzyme (Beijing Total Gold Biotechnology Co., Ltd., Beijing, China) were used to amplify the gene fragments at the optimal primer annealing temperature. A fully automated enzyme labeling instrument (Thermo Fisher Scientific) was used to determine the CXCR4, SDF-1, IL-1β, TNF-α, IL-6, and NF-κB mRNA levels as well as the A260/280 ratio. Agarose electrophoresis buffer (LABJIC Biotechnology Co., Ltd., Beijing, China) was used to prepare a 1% agarose gel to determine the RNA concentration.

2.7. Real-Time Quantitative Polymerase Chain Reaction (qPCR)

Each reaction contained 0.4 μl of upstream primer, 0.4 μl of related primer, 0.8 μl of template, 10 μl of 2×PerfectStart Green qPCR SuperMix (Beijing Quan Shi Jin Biotechnology Co., Ltd., Beijing, China), 0.4 μl of Passive Reference Dye (50×), and 8 μl of nuclease-free water to a total volume of 20 μl. Seven pairs of primers were designed by Sangyo Bioengineering Co., Ltd. (Shanghai, China); the primer names, primer sequences, gene IDs, and primer lengths are shown in Table 2. The experimental conditions were 94°C for 30 s, 59.6°C for 15 s, and 72°C for 10 s (44 cycles). The experimental results were obtained by repeating the experiment three times and taking the average value. The mRNA expression of the target genes CXCR4, SDF-1, IL-1β,

TNF-α, IL-6 and NF-κB was calculated by the $2^{-\Delta\Delta CT}$ method using glyceraldehyde dehydrogenase 3-phosphate (GAPDH) as the internal reference gene (n=6).

Table 2. Primer sequences, gene numbers and product amplification lengths

Gene	Primer sequences	Product length (bp)	Gene ID
GAPDH	F: GAGGAGGCATTGCTGATGAT	20	NM_002046.7
	R: GAAGGCTGGGGCTCATTT	18	
CXCR4	F: CTCCTCTTTGTCATCACGCTTCC	23	NM_001008540
	R: GGATGAGGACACTGCTGTAGAG	22	
SDF-1	F: GGGAAGACCCGTGTTACCAG	20	NM_199168.4
	R: AGTCCAGCCTGCTATCCTCA	20	
NF-κB	F: TGTGTTTGTCCAGCTTCG	18	NM_001165412.2
	R: GCTTCTGACGTTTCCTCTG	19	
IL-1β	F: TCGCCAGTGAAATGATGGCT	20	NM_000576
	R: TGAAGCCCTTGCTGTAGTGG	20	
IL-6	F: GAGGAGACTTGCCTGGTGAA	20	NM_000600.5

	R: CAGCTCTGGCTTGTTCCTCA	20	
TNF-α	F: ATGAGCACTGAAAGCATGATCC	22	NM_000594.4
	R: AGGAGAAGAGGCTGAGGAACAAG	23	

2.8. Statistical Analysis

Excel was used for data entry and the development of statistical tables, GraphPad Prism 8.0.2 was used to generate statistical graphs, and SPSS 22.0 was used to statistically analyze the experimental and control groups; a t test was used for data that were normally distributed and exhibited homogeneity of variance, and the Mann–Whitney test was used for data that did not satisfy these conditions. Measurement data are expressed as the mean ± standard deviation (± s), test level $\alpha = 0.05$, and $P < 0.05$ indicated significance.

3. Results

3.1. Effects of Different Exposure Times and Sodium Fluoride Concentrations on the Survival of Human LX-2 Cells

We first investigated the effects of different concentrations of sodium fluoride and different fluoride staining times on cell survival by using CCK-8 assays. The results showed that with different concentrations (0, 0.5, 1, 2, 4 and 8 mmol/l) of NaF, the survival rate of the cells gradually decreased with increasing fluoride concentration, and the LD50 values for LX-2 cells at 12 h, 24 h and 48 h were all approximately 2 mmol/l NaF (see Figure 1). Therefore, 2 mmol/l NaF was selected as the treatment concentration for the subsequent experiments.

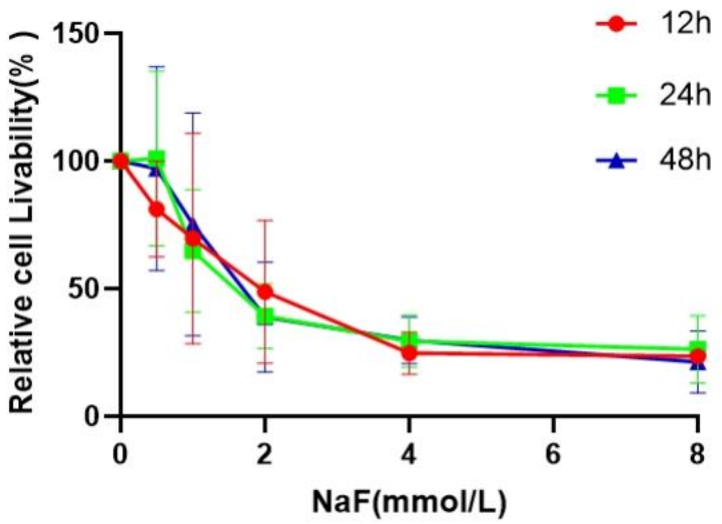

Figure 1. Effects of sodium fluoride exposure time and concentration on the viability of LX-2 cells determined by CCK-8 assays (*mean*± *SE*, *n*=4).

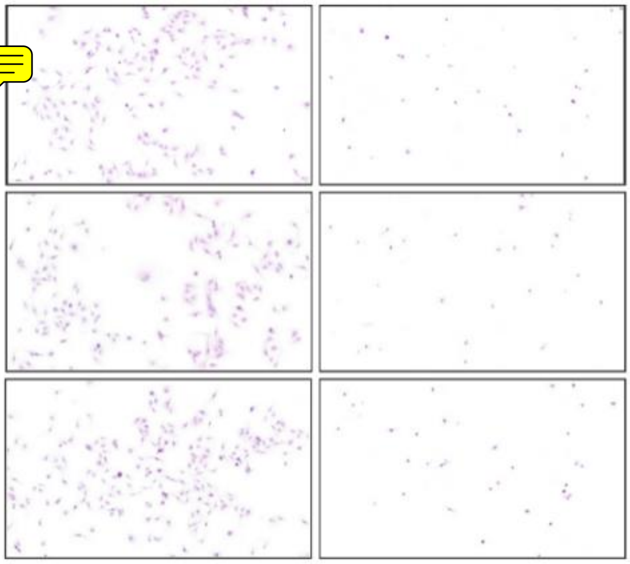

Figure 2. Results of HE staining of cells (*n*=3)

The above figure shows the morphology of normal hepatocytes and sodium fluoride-treated LX-2 hepatocytes under a light microscope. Sodium fluoride was added at a concentration of 2 mmol/l for a period of 24 h. The cells in the fluoride-treated group were

relatively rounded or swollen; moreover, the nuclei were more centralized, and the cytoplasm was dramatically smaller in volume. Finally, the cellular arrangement was disorganized, and the interstitial space between the cells was enlarged, among other phenomena (see Figure 2).

3.2. Increased Expression of Inflammatory Factors in the Supernatants of NaF-Treated Hepatocytes

The OD values of the experimental group and the control group were determined at 450 nm by an enzyme marker, and *t* tests were used to identify any difference between the OD values of the two groups. ELISA results showed that the expression of CXCR4, SDF-1, IL-1β, TNF-α, IL-6 and NF-κB-specific proteins was significantly increased in the fluoride-treated cell group compared to the control group ($P < 0.05$), as shown in Table 3 and Figure 3.

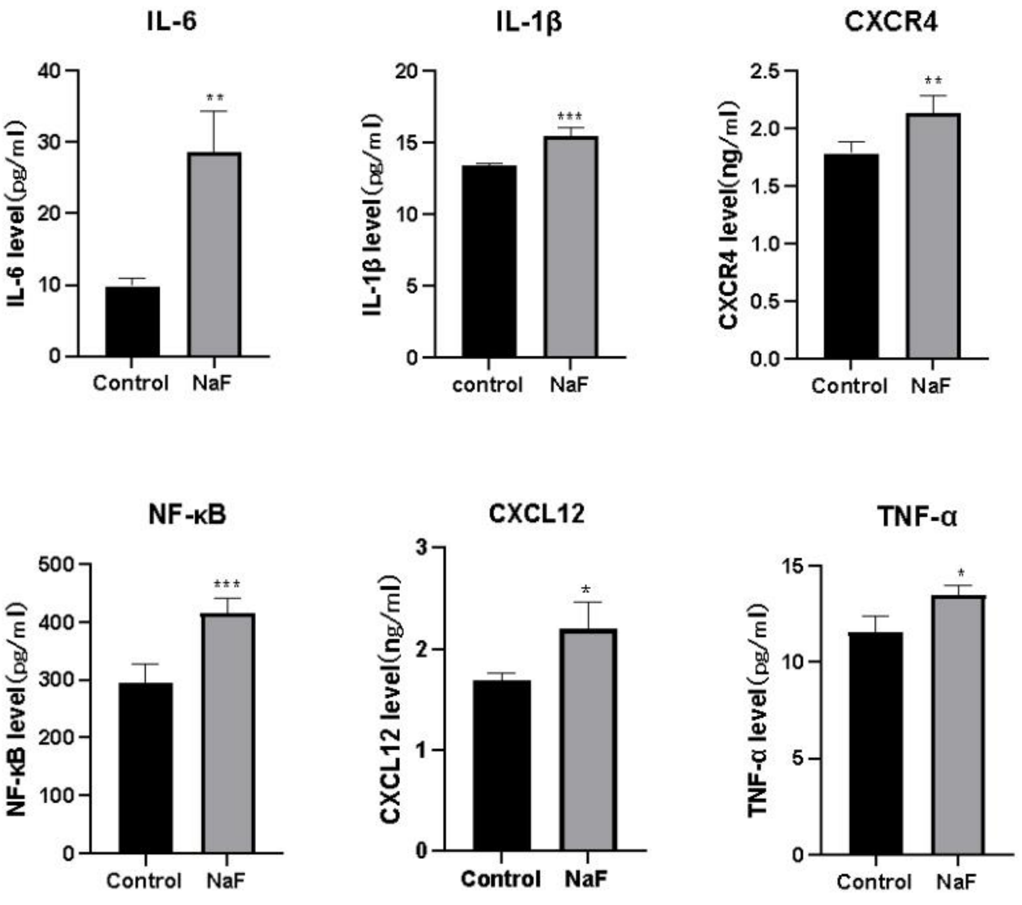

Figure 3. Expression level of each target protein in the supernatants of the fluoride-treated and control cells (*mean±SE*, n=4). $^*P < 0.05$, $^{**}P < 0.01$, and $^{***}P < 0.001$ compared with the control group.

Table 3. Expression [illegible]ls of CXCR4, IL-1β, IL-6, SDF-1, TNF-α and NF-κB in Cell Supernatants of the Fluoride-Treated and Control Groups ($\bar{x} \pm s$)

Testing indicators	CXCR4 (ng/ml)	IL-1β (pg/ml)	IL-6 (pg/ml)	SDF-1 (ng/ml)	TNF-α (pg/ml)	NF-κB (pg/ml)
Number of samples in the fluoride group (*n*)	4	4	4	4	4	4
Sample size of the control group (*n*)	4	4	4	4	4	4
Indicator expression in the fluoride-treated groups	2.13±0.07[a]	15.5±0.29[a]	28.61±2.55[a]	2.2±0.12[a]	13.52±0.27[a]	415.40±14.72[a]
Expression of indicator in the control group	1.79±0.04	13.37±0.09	9.84±0.49	1.68±0.04	11.55±0.51	295.28±11.31
t	4.30	6.97	7.24	4.17	3.40	6.47

P	<0.05	<0.05	<0.05	<0.05	<0.05	<0.05

Note: [a] $P < 0.05$ compared to the same indicator in the control group, *t* value is from a two-sample *t* test, n=4

3.3. Increased Expression of Inflammatory Factors in NaF-Treated Hepatocyte Extracts

ELISAs of cell extracts showed that CXCR4, SDF-1 (CXCL12), IL-1β, TNF-α, IL-6, and NF-κB protein expression was significantly increased in the fluoride-treated cell group compared to the control group ($P < 0.05$), as shown in Table 4 and F[illegible]re 4.

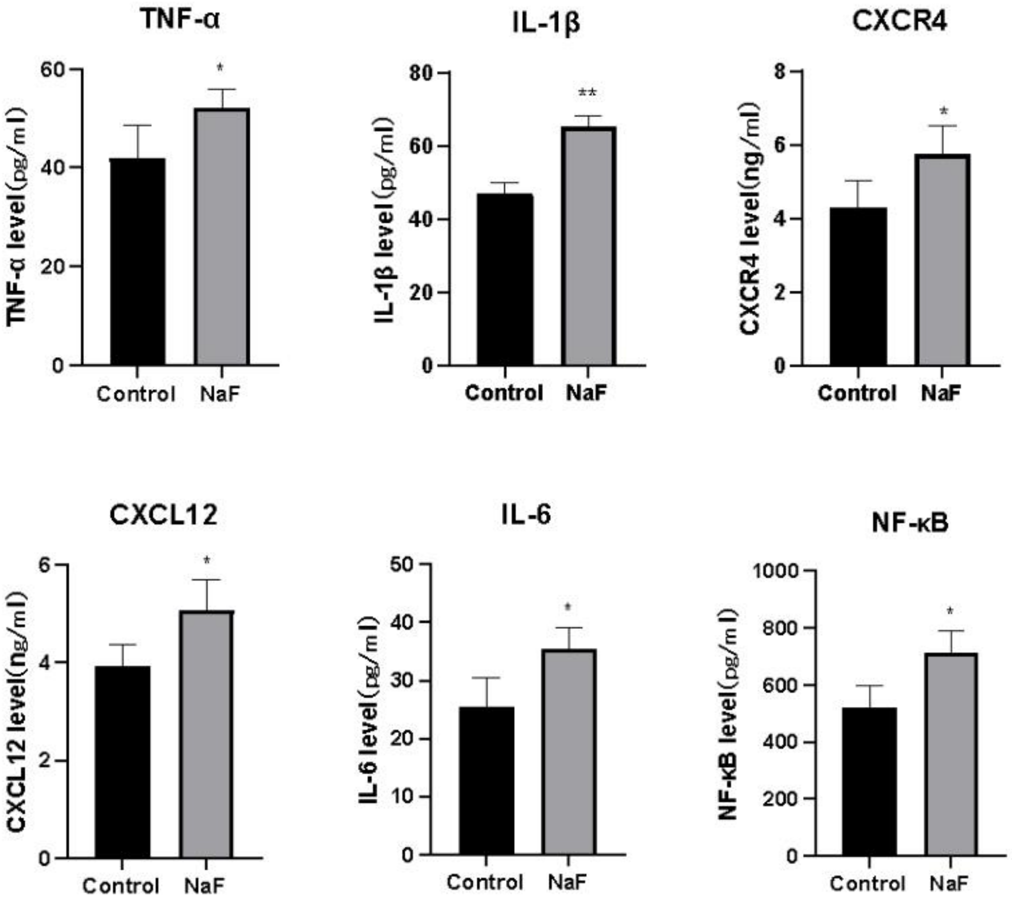

Figure 4. Expression level of each target protein in cell extracts from the fluoride-treated and control cells (*mean*±*SE*, *n*=4). **P* < 0.05 and ***P* < 0.01 compared with the control group.

Table 4. Expression levels of SDF-1, TNF-α, NF-κB, CXCR4, IL-1β and IL-6 in Cell Extracts of the Fluoride-Treated and Control Groups ($\bar{x}$ ±s)

Testing indicators	SDF-1 (ng/ml)	TNF-α (pg/ml)	NF-κB (pg/ml)	CXCR4 (ng/ml)	IL-1β (pg/ml)	IL-6 (pg/ml)
Number of samples in the fluoride group (*n*)	4	4	4	4	4	4
Sample size of the control group (*n*)	4	4	4	4	4	4
Indicator expression in the fluoride-contaminated groups	5.08±0.32[a]	41.99±1.91[a]	713.18±39.38[a]	5.74±0.38[a]	65.19±1.76[a]	35.49±1.82[a]
Expression of indicator in the control group	3.94±0.21	52.10±3.33	523.17±36.27	4.28±0.37	46.93±1.80	25.47±2.50
t	3.00	2.64	3.55	2.73	7.23	3.25

P	<0.05	<0.05	<0.05	<0.05	<0.05	<0.05

Note: [a] $P < 0.05$ compared to the same indicator in the control group, *t* value is a two-sample *t* test, n=4

Cell extracts were mainly obtained by repeated freezing and thawing of the ce[illegible]nd the lysates were centrifuged to determine whether inflammatory factor receptors were expressed in the cells. Cell supernatants were directly centrifuged and used to determine whether inflammatory factor receptors were expressed on the surface of the cell membranes. ELISAs of both the cell extracts and the cell supernatants showed that fluoride significantly increased the expression of cellular inflammatory factors at this conce[illegible]ion.

3.4. Analysis of qPCR Results

Fluorescent dyes were added to the PCR system, and fluorescent signal accumulation was used to monitor the PCR process. We used GAPDH as the internal reference gene, and the 2-ΔΔCT method was used to calculate the mRNA levels of CXCR4, SDF-1, IL-1β, TNF-α, IL-6 and NF-κB, which were determined by qPCR in the fluoride group and the control group, as shown in Figure 5.

We concluded that fluoride enhanced the SDF-1/CXCR4 signaling axis and the inflammatory factors IL-1β, TNF-α, IL-6 and NF-κB in normal hepatocytes, both in terms of gene expression and protein expression, as shown by ELISAs and qPCR.

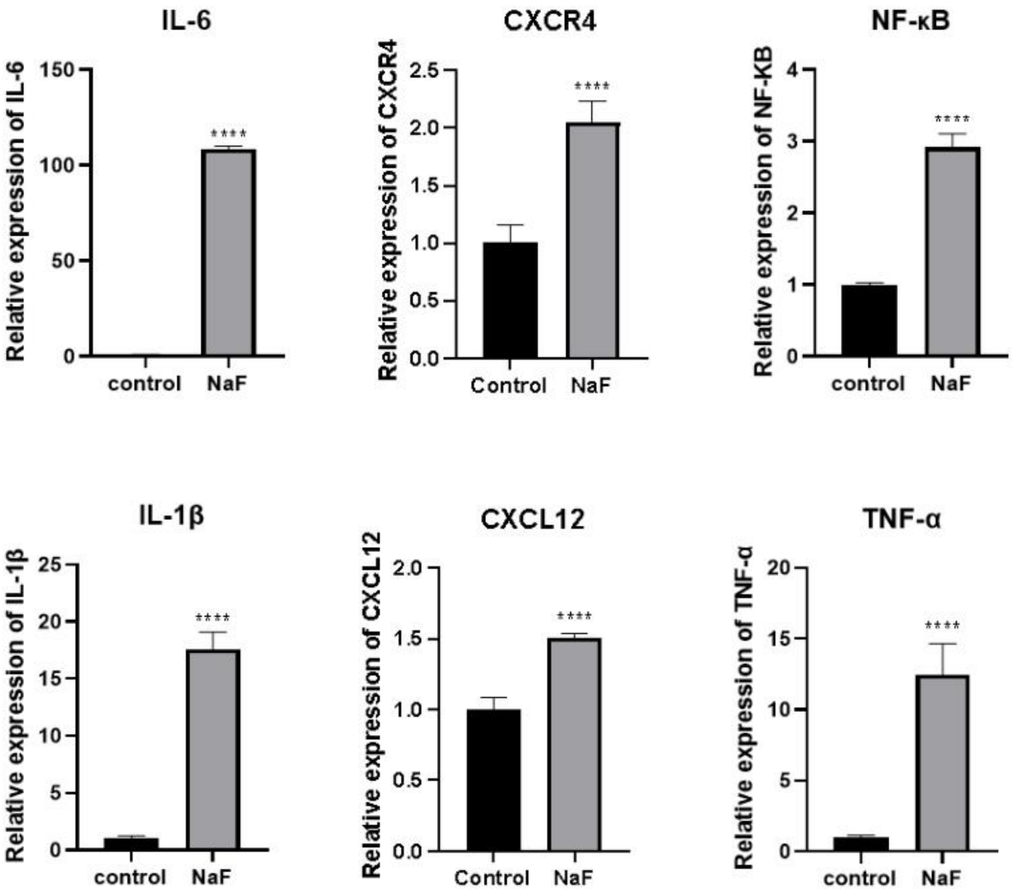

Figure 5. mRNA expression levels of indicators in the fluoride group and the control group (*****P* < 0.001, *mean*±SE, *n*=6).

4. Discussion

Fluorosis occurs in many regions of the world and affects human quality of life to a certain extent, not only by placing an economic burden on families but also by impeding the economic development of a region [39]. However, the pathogenesis of fluorosis has not yet been clarified, although it is now known that fluorosis mainly involves oxidative stress, endoplasmic reticulum stress, and signaling pathways that damage human tissues or organs [40-42]. Although inflammatory damage is not widely recognized as a major component of fluorosis,

fluorosis has been shown to cause increased expression of inflammatory factors such as IL-6 [43,44]. Thus, inflammatory damage may be one of the pathogenic mechanisms leading to fluorosis, but whether it plays a role in specific cases needs to be evaluated on a case-by-case basis. SDF-1/CXCR4, as an important signaling pathway, recruits cellular immune factors to participate in the body's inflammatory response [45-46]. In this study, human LX-2 cells were treated with different concentrations of NaF in v[illegible], and the cell survival rate was detected by the CCK-8 method. The LD50 value, which was found to be 2 mmol/l after fluor[illegible]ion with different concentrations of NaF (0.5, 1, 2, 4, and 8 mmol/l) for 12, 24, and 48 h, was selected as the appropriate concentration for further experiments. We analyzed the morphology of the cells before and after fluoride treatment, as well as the mRNA and protein expression of each target, CXCR4, CXCL12, NF-κB, IL-6, IL-1β and TNF-α. HE staining revealed that the cells in the fluoride-treated group exhibited a rounded or swollen morphology, with more compact nuclei; moreover, the arrangement of the cells was disorganized, and the intercellular gap was enlarged. HE staining showed that excessive fluorine caused a certain degree of damage to normal human hepatocytes. ELISAs showed that the levels of CXCR4, CXCL12, NF-κB, IL-6, IL-1β and TNF-α in the cell supernatants and cell extracts were significantly elevated in the experimental group compared with the control group ($P<0.05$). qPCR was used to determine the mRNA levels of the target genes CXCR4, CXCL12, NF-κB, IL-6, IL-1β and TNF-α. TNF-α mRNA expression levels were significantly increased in the experimental group compared with the control group ($P < 0.05$), consistent with the ELISA res[illegible].

In the present study, one limitation is that we did not explore the relationship between the signaling axis and inflammatory factors, which needs to be explored by signaling pathway

antagonist experiments. However, the present cellular experiments successfully identified the SDF-1/CXCR4 signaling axis, and the related inflammatory factors NF-κB, IL-6, IL-1β, and TNF-α were expressed at high levels in hepatocytes treated with high concentrations of fluoride. We found that the gene and protein expression levels of SDF-1/CXCR4 signaling axis members in the fluoride group were significantly higher than those in the blank control group, and the mRNA expression levels of inflammatory molecules such as NF-κB, IL-6, IL-1β, and TNF-α were consistent with those of the SDF-1/CXCR4 signaling axis in both groups. We speculated that fluoride could lead to upregulated expression of genes and proteins of the SDF-1/CXCR4 signaling axis in hepatocytes and then activate the NF-κB signaling pathway to release excessive inflammatory factors such as IL-6, IL-1β and TNF-α. The association between the pathogenesis of inflammation in fluorosis and signaling pathways may be confirmed by further investigating signaling pathway antagonists that block signaling pathways to explore the expression of inflammatory factors. As the factors that cause inflammation are very complex, the present findings may also suggest the synergistic activation of the NF-κB signaling pathway and other signaling pathways, which are involved in the release of the inflammatory factors IL-6, IL-1β, and TNF-α [48]. The mechanism needs to be further studied.

5. Conclusion

Excessive sodium fluoride induced an increase in the expression levels of the hepatic cellular inflammatory factors IL-6, TNF-α and IL-1β along with the chemokine signaling axis SDF-1/CXCR4 and the inflammatory signaling pathway NF-κB.

Author Contributions: Conceptualization, R.Y.; methodology, R.Y.; software, R.Y.; validation, R.Y. and H.S.; formal analysis, R.Y. and H.S.; investigation, R.Y., G.P. and R.Y.; data curation, Y.L., J.H. and M.W.; writing—original draft preparation, R. Y; writing—review and editing, R.Y.; visualization, R.Y., X.C. and P.C. and J.M. and L.Q.; supervision, R.Y. and Y.Z. and S.Z.; project administration, Q.Z.; funding acquisition, Q.Z. All authors have read and agreed to the published version of the manuscript.

Funding: The Second Comprehensive Scientific Investigation and Research Support of Qinghai Tibet Plateau, grant number 2019QZKK0607.

Data Availability Statement: Not applicable.

Acknowledgments: The authors thank the Institute of Geographic Sciences and Resources of the Chinese Academy of Sciences for financial support.

Conflicts of Interest: The authors declare no conflicts of interest.

References

1 Mekler, L. On the Problem of Oncogene of Tumour Viruses. *Acta Virol.* **1975**, *19*, 501–508.

2 Bronckers, A. L. J. J.; Lyaruu, D. M.; DenBesten, P. K. The Impact of Fluoride on Ameloblasts and the Mechanisms of Enamel Fluorosis. *J. Dent. Res.* **2009**, *88*, 877–893.

3 Zhu, Z. J.; Yu, Y. N.; Chen, R.; et al. Role of Hedgehog Signaling Pathway on Cartilage Tissue Damage in Chronic Fluorosis Rats. *Chin. J. Public Health* **2018**, *34*, 241–245.

4 Li, Y.; Zhang, H.; Chen, B.; et al. Influence of Brewing Method of Brick Tea on the Leaching Concentration of Tea Fluoride. *Health Research* **2012**, *41*, 2.

5 Li, C.; Mong, Y.; Jiang, C. Epidemiologic Status of Geofluorosis in Mainland China. *Pract. Prev. Med.* **2008**, *15*, 4.

6 Jiang, S.; Chen, Q.; Zhang, H. Overview of the Treatment Research of Endemic Fluorosis in the Past 10 Years. *Chinese Journal of Endemic Disease Control* **2020**, *35*, 2.

7 Zhang, C. Determination of Plasma Vitamin C and Serum Vitamin E in Patients with Osteofluorosis. *Chinese Journal of Endemic Disease Control* **1990**.

8 Huang, L.; Chen, X. Experimental Study on the Changes of Several Trace Elements during the Healing Period of Fractures with Fluorosis: A Study of Silicate. *Chinese Journal of Endemic Disease Control* **1993**, *8*, 204–207.

9 Xiao, Q.; Li, F.; Wang, J.; et al. Exploration of the Effects of Fluorosis and the Long-Term Effects of Bone after Detachment from a High Fluoride Source in a Severely Ill Village with Coal-Baked Corn Fluorosis in Shucheng County, Guizhou. *Contemporary Medicine* **2016**, *22*, 3.

10 Onipe, T.; Edokpayi, J. N.; Odiyo, J. O. A Review on the Potential Sources and Health Implications of Fluoride in Groundwater of Sub-Saharan Africa. *J. Environ. Sci. Health Part A* **2020**, *55*, 1078–1093.

11 Valadas, L. A. R.; Girão Jr, F. J.; Lotif, M. A. L.; Fernández, C. E.; Bandeira, M. A. M.; Fonteles, M. M. D. F.; Bottenberg, P.; Squassi, A. Fluoride Concentration in Teas Derived from Camellia Sinensis Produced in Argentina. *Environ. Monit. Assess.* **2022**, *194*, 682.

12 Choubisa, S. L.; Choubisa, D.; Choubisa, A. Fluoride Contamination of Groundwater and Its Threat to Health of Villagers and Their Domestic Animals and Agriculture Crops in Rural Rajasthan, India. *Environ. Geochem. Health* **2022**, *45*, 607–628.

13 Qiang, L.; Zhao, Z. J. Prevalence of Brick Tea-Type Fluorosis in Children Aged 8-12 Years in Qinghai Province, China. *Biomed. Environ. Sci.* **2021**, *34*, 334–336.

14 Jandu, J.; Dzyuba, N.; Barry, S. White Enamel Lesions in Children: Considering Caries, Chronological and Congenital Causes and Their Consequent Care. *Br. Dent. J.* **2021**, *230*, 523–527.

15 Karamian, O.; Kozhanov, V.; Chmykhova, N. Mechanisms of Motor Neuron Interaction in the Isolated Spinal Cord of Rat Pups. *Neurophysiology* **1988**, *20*, 243–250.

16 Morris, A. J.; O´Connor, R.; Holmes, R.; Landes, D.; Shah, K.; Tanday, A.; Vernazza, C. Dental Fluorosis. *Br. Dent. J.* **2022**, *232*, 492.

17 Efe, U.; Dede, S.; Yüksek, V.; Çetin, S. Apoptotic and Oxidative Mechanisms in Liver and Kidney Tissues of Sheep with Fluorosis. *Biol. Trace Elem. Res.* **2021**, *199*, 136–141.

18 Wang, Q.; Meng, Q.; Meng, J. Minimally Invasive Esthetic Management of Dental Fluorosis: A Case Report. *J. Int. Med. Res.* **2020**, *48*, 0300060520967538.

19 Quadri, J. A.; Sarwar, S.; Pinky; Kar, P.; Singh, S.; Mallick, S. R.; Arava, S.; Nag, T. C.; Roy, T. S.; Shariff, A. Fluoride Induced Tissue Hypercalcemia, IL-17 Mediated Inflammation and Apoptosis Lead to Cardiomyopathy: Ultrastructural and Biochemical Findings. *Toxicology* **2018**, *406-407*, 44–57.

20 Zhao, Y.; Yu, Y.; Ommati, M. M.; Xu, J.; Wang, J.; Zhang, J.; Sun, Z.; Niu, R.; Wang, J. Multiomics Analysis Revealed the Molecular Mechanism of miRNAs in Fluoride-Induced Hepatic Glucose and Lipid Metabolism Disorders. *J. Agric. Food Chem.* **2022**, *70*, 14284–14295.

21 Zhang, Z.; Zhou, B.; Wang, H.; Wang, F.; Song, Y.; Liu, S.; Xi, S. Maize Purple Plant Pigment Protects against Fluoride-Induced Oxidative Damage of Liver and Kidney in Rats. *Int. J. Environ. Res. Public Health* **2014**, *11*, 1020–1033.

22 Zhao, S.; Guo, J.; Xue, H.; Meng, J.; Xie, D.; Liu, X.; Yu, Q.; Zhong, H.; Jiang, P. Systematic Impacts of Fluoride Exposure on the Metabolomics of Rats. *Ecotoxicol. Environ. Saf.* **2022**, *242*, 113888.

23 Wang, W.; Ha, C.; Lin, T.; Wang, D.; Wang, Y.; Gong, M. Celastrol Attenuates Pain and Cartilage Damage via SDF-1/CXCR4 Signalling Pathway in Osteoarthritis Rats. *J. Pharm. Pharmacol.* **2018**, *70*, 81–88.

24 Ngamsri, K. C.; Jans, C.; Putri, R. A.; Schindler, K.; Gamper-Tsigaras, J.; Eggstein, C.; Köhler, D.; Konrad, F. M. Inhibition of CXCR4 and CXCR7 is Protective in Acute Peritoneal Inflammation. *Front. Immunol.* **2020**, *11*, 407.

25 Liu, Z. C.; Wang, Z. L.; Huang, C. Y.; Fu, Z. J.; Liu, Y.; Wei, Z. C.; Liu, S. G.; Ma, C.; Shen, J. L.; Duan, D. D. Duhuo Jisheng Decoction Inhibits SDF-1-Induced Inflammation and Matrix Degradation in

Human Degenerative Nucleus Pulposus Cells In Vitro through the CXCR4/NF-κB Pathway. *Acta Pharmacol. Sin.* **2018**, *39*, 912–922.

26 Wang, S.; Gao, S.; Li, Y.; Qian, X.; Luan, J.; Lv, X. Emerging Importance of Chemokine Receptor CXCR4 and Its Ligand in Liver Disease. *Frontiers in Cell and Developmental Biology* **2021**, *9*, 716842.

27 Mousavi, A. CXCL12/CXCR4 Signal Transduction in Diseases and Its Molecular Approaches in Targeted-Therapy. *Immunol. Lett.* **2020**, *217*, 91–115.

28 Zhou, Y.; Cao, H. B.; Li, W. J.; Zhao, L. The CXCL12 (SDF-1)/CXCR4 Chemokine Axis: Oncogenic Properties, Molecular Targeting, and Synthetic and Natural Product CXCR4 Inhibitors for Cancer Therapy. *Chin. J. Nat. Med.* **2018**, *16*, 801–810.

29 Zhao, Y.; Pu, G.; Li, Y.; Jiang, H.; Zhang, Q.; Chen, P.; Lu, Q.; Wang, M.; Yang, R. Serum Levels of CXCR4, SDF-1, MCP-1, NF-κB and ERK1/2 in Patients with Skeletal Fluorosis. *Int. J. Environ. Res. Public Health* **2022**, *19*, 16555.

30 Barnabei, L.; Laplantine, E.; Mbongo, W.; Rieux-Laucat, F.; Weil, R. NF-κB: At the Borders of Autoimmunity and Inflammation. *Front. Immunol.* **2021**, *12*, 716469.

31 Alexandrov, P.; Zhao, Y.; Li, W.; Lukiw, W. Lipopolysaccharide-Stimulated, NF-kB-, miRNA-146a-and miRNA-155-Mediated Molecular-Genetic Communication between the Human Gastrointestinal Tract Microbiome and the Brain. *Folia Neuropathol.* **2019**, *57*, 211–219.

32 Sun, E.; Motolani, A.; Campos, L.; Lu, T. The Pivotal Role of NF-kB in the Pathogenesis and Therapeutics of Alzheimer's Disease. *Int. J. Mol. Sci.* **2022**, *23*, 8972.

33 Lin, Y.; Ma, Q.; Li, L.; Wang, H. The CXCL12–CXCR4 Axis Promotes Migration, Invasiveness, and EMT in Human Papillary Thyroid Carcinoma B-CPAP Cells via NF-κB Signaling. *Biochem. Cell Biol.* **2018**, *96*, 619–626.

34 Si, M.; Ma, Z.; Zhang, J.; Li, X.; Li, R.; Wang, C.; Jia, H.; Luo, S. Qingluoyin Granules Protect against Adjuvant-Induced Arthritis in Rats via Downregulating the CXCL12/CXCR4-NF-κB Signalling Pathway. *Pharm. Biol.* **2021**, *59*, 1439–1449.

35 Khalighfard, S.; Khori, V.; Esmati, E.; Ahmadi, F.; Amiriani, T.; Poorkhani, A.; Sadani, S.; Khodayari, S.; Khodayari, H.; Kalhori, M. R.; Keshavarz, P.; Alizadeh, A. M. Breast Tumor Metastasis Following Filgrastim Administration Due to the SDF-1/CXCR4 Pathway. *Med. Oncol.* **2023**, *40*, 74.

36 Hu, Q.; Zhang, W.; Wu, Z.; Tian, X.; Xiang, J.; Li, L.; Li, Z.; Peng, X.; Wei, S.; Ma, X.; Zhao, Y. Baicalin and the Liver-Gut System: Pharmacological Bases Explaining Its Therapeutic Effects. *Pharmacol. Res.* **2021**, *165*, 105444.

37 Ablamunits, V.; Lepsy, C. Blocking TNF Signaling May Save Lives in COVID-19 Infection. *Mol. Biol. Rep.* **2022**, *49*, 2303–2309.

38 Zheng, H. C.; Xue, H.; Jiang, H. M. The Roles of ING5 in Cancer: A Tumor Suppressor. *Frontiers in Cell and Developmental Biology* **2022**, *10*, 1012179.

39 Srivastava, S.; Flora, S. J. S. Fluoride in Drinking Water and Skeletal Fluorosis: A Review of the Global Impact. *Curr. Environ. Health Rep.* **2020**, *7*, 140–146.

40 Qiao, L.; Liu, X.; He, Y.; Zhang, J.; Huang, H.; Bian, W.; Chilufya, M. M.; Zhao, Y.; Han, J. Progress of Signaling Pathways, Stress Pathways and Epigenetics in the Pathogenesis of Skeletal Fluorosis. *Int. J. Mol. Sci.* **2021**, *22*, 11932.

41 Nagendra, A. H.; Bose, B.; Shenoy, P. S. Recent Advances in Cellular Effects of Fluoride: An Update on Its Signalling Pathway and Targeted Therapeutic Approaches. *Mol. Biol. Rep.* **2021**, *48*, 5661–5673.

42 Babu, S.; Manoharan, S.; Ottappilakkil, H.; Perumal, E. Role of Oxidative Stress-Mediated Cell Death and Signaling Pathways in Experimental Fluorosis. *Chem. Biol. Interact.* **2022**, *365*, 110106.

43 Waugh, D. T. Fluoride Exposure Induces Inhibition of Sodium/Iodide Symporter (NIS) Contributing to Impaired Iodine Absorption and Iodine Deficiency: Molecular Mechanisms of Inhibition and Implications for Public Health. *Int. J. Environ. Res. Public Health* **2019**, *16*, 1086.

44 Waugh, D. T. The Contribution of Fluoride to the Pathogenesis of Eye Diseases: Molecular Mechanisms and Implications for Public Health. *Int. J. Environ. Res. Public Health* **2019**, *16*, 856.

45 Li, X.; Lan, X.; Zhao, Y.; Wang, G.; Shi, G.; Li, H.; Hu, Y.; Xu, X.; Zhang, B.; Ye, K.; Gu, X.; Du, C.; Wang, H. SDF-1/CXCR4 Axis Enhances the Immunomodulation of Human Endometrial Regenerative Cells in Alleviating Experimental Colitis. *Stem Cell Res. Ther.* **2019**, *10*, 204.

46 Tunc, T.; Cekmez, F.; Cetinkaya, M.; Kalayci, T.; Fidanci, K.; Saldir, M.; Babacan, O.; Sari, E.; Erdem, G.; Cayci, T.; Kul, M.; Kavuncuoglu, S. Diagnostic Value of Elevated CXCR4 and CXCL12 in Neonatal Sepsis. *The Journal of Maternal-Fetal & Neonatal Medicine* **2015**, *28*, 356–361.

47 Chen, L., Kuang, P., Liu, H., Wei, Q., Cui, H., Fang, J., …Zhao, L.(2019). Sodium Fluoride (NaF) Induces Inflammatory Responses Via Activating MAPKs/NF-κB Signaling Pathway and Reducing Anti-inflammatory Cytokine Expression in the Mouse Liver. Biol Trace Elem Res 189, 157–171. https://doi.org/10.1007/s12011-018-1458-z.
