# Supplementary material for: Expression of SDF-1/CXCR4 and related inflammatory factors in sodium fluoride-treated hepatocytes
Source: PLoS One. 2024 Jun 21;19(6):e0302530. doi: 10.1371/journal.pone.0302530 (PMC11192373; doi:10.1371/journal.pone.0302530)
Supplement: Attachment — Submitted filename: Comments.docx [file pone.0302530.s003.docx]

The authors have studied the “Expression of SDF-1/CXCR4 and Related Inflammatory Factors in Sodium Fluoride-Treated Hepatocytes”. This research article is well-designed and interesting. The manuscript is suitable for publication but there are minor technical flaws which need to be addressed. Some of these concerns are mentioned below:

1. Why were just LX-2 astrocytes used in the study? Briefly explain it.
2. The manuscript does not include a reference to the cell culture methodology used.
3. The apparatus used for microscopy throughout the study is not mentioned.
4. The source from which the primers were chosen is unavailable. Give the source in manuscript.
5. There is a lack of referencing in the entire methodology. For the methods section, not a single reference is provided.
6. In photography, the scale is not indicated.
7. It is advised that a higher magnification image of HE staining of cells be included because there is no evident comparison with the images provided with the current file.
8. The outcome is repeated in the discussion section. It should be modified.
9. The significance of the current investigation is not clearly stated in the text. What is the relevance of the study? Describe it.
10. Is there any recent research on fluorosis with inflammation? If yes, explain why the current investigation is necessary.
