# Supplementary material for: Expression of SDF-1/CXCR4 and related inflammatory factors in sodium fluoride-treated hepatocytes
Source: PLoS One. 2024 Jun 21;19(6):e0302530. doi: 10.1371/journal.pone.0302530 (PMC11192373; doi:10.1371/journal.pone.0302530)
Supplement: Attachment — Submitted filename: Response to reviewers.docx [file pone.0302530.s004.docx]

The authors have studied the “Expression of SDF-1/CXCR4 and Related Inflammatory Factors in Sodium Fluoride-Treated Hepatocytes”. This research article is well-designed and interesting. The manuscript is suitable for publication but there are minor technical flaws which need to be addressed. Some of these concerns are mentioned below:

1. Why were just LX-2 astrocytes used in the study? Briefly explain it.

Firstly, LX-2 cells are easy to culture, grow vigorously and require less conditions such as culture medium, and secondly, the inflammatory factors as well as signaling axes involved in this study are less studied in LX-2 cells, which is of great significance for the study of the liver system due to fluorosis.

2. The manuscript does not include a reference to the cell culture methodology used.

I have already mentioned the cell culture process in Materials and Methods under Cell Culture and Transmission

3. The apparatus used for microscopy throughout the study is not mentioned.

The microscope mentioned in the article is a general optical microscope, which is used directly for observing the state of cell growth as well as HE sections, and I don't feel that the article needs to go into further detail about the specific instrumentation involved in that general microscope. Perhaps I misunderstood what the author meant by specific instruments, I don't quite understand it!

4. The source from which the primers were chosen is unavailable. Give the source in manuscript.

I have already mentioned in my manuscript that my primers are from Shanghai Sangong Bioengineering Co. in China

5. There is a lack of referencing in the entire methodology. For the methods section, not a single reference is provided.

The ELISA, PCR, HE staining, etc. covered in the article I don't feel the need to provide appropriate references for this, they are just one method

6. In photography, the scale is not indicated.

I have labeled the HE stained images with the scale you requested.

7. It is advised that a higher magnification image of HE staining of cells be included because there is no evident comparison with the images provided with the current file.

Dear Sir, I have tried to make the cell HE staining pictures as clear as possible, mainly by comparing the number of cells and cell morphology of the two groups, thank you for your valuable comments!

8. The outcome is repeated in the discussion section. It should be modified.

This section is currently redacted.

9. The significance of the current investigation is not clearly stated in the text. What is the relevance of the study? Describe it.

The introduction and conclusion of the article have actually explained the significance of the study, mainly through the sodium fluoride infiltration of human LX-2 hepatocytes, and then explore the signaling pathway and the expression level of related inflammatory factors, in order to provide ideas for the further exploration of fluorosis-induced pathogenesis of the hepatic system

10. Is there any recent research on fluorosis with inflammation? If yes, explain why the current investigation is necessary.

The recognized doctrines of fluorosis are oxidative stress, signaling pathways and other doctrines. The theory of inflammatory response to fluorosis has not been established, and domestic and foreign research is still in the exploratory stage, with relatively little research content. Therefore, it is slightly important to study the inflammatory response from the perspective of the liver pathogenesis of fluorosis to provide new ideas for the prevention and treatment of fluorosis.
